# Pioglitazone administration alters ovarian gene expression in aging obese lethal yellow mice

**DOI:** 10.1186/1477-7827-6-10

**Published:** 2008-03-18

**Authors:** John D Brannian, Kathleen M Eyster, Mitch Weber, Maureen Diggins

**Affiliations:** 1 Department of Obstetrics and Gynecology and Division of Basic Biomedical Sciences Sanford School of Medicine of The University of South Dakota, Sioux Falls and Vermillion, SD, USA; 2 Sanford Research USD, Sioux Falls, SD, USA; 3 Department of Biology, Augustana College, Sioux Falls, SD, USA

## Abstract

**Background:**

Women with polycystic ovary syndrome (PCOS) are often treated with insulin-sensitizing agents, e.g. thiazolidinediones (TZD), which have been shown to reduce androgen levels and improved ovulatory function. Acting via peroxisome proliferator-activated receptor (PPAR) gamma, TZD alter the expression of a large variety of genes. Lethal yellow (LY; C57BL/6J Ay/a) mice, possessing a mutation (Ay) in the agouti gene locus, exhibit progressive obesity, reproductive dysfunction, and altered metabolic regulation similar to women with PCOS. The current study was designed to test the hypothesis that prolonged treatment of aging LY mice with the TZD, pioglitazone, alters the ovarian expression of genes that may impact reproduction.

**Methods:**

Female LY mice received daily oral doses of either 0.01 mg pioglitazone (n = 4) or an equal volume of vehicle (DMSO; n = 4) for 8 weeks. At the end of treatment, ovaries were removed and DNA microarrays were used to analyze differential gene expression.

**Results:**

Twenty-seven genes showed at least a two-fold difference in ovarian expression with pioglitazone treatment. These included leptin, angiopoietin, angiopoietin-like 4, Foxa3, PGE1 receptor, resistin-like molecule-alpha (RELM), and actin-related protein 6 homolog (ARP6). For most altered genes, pioglitazone changed levels of expression to those seen in untreated C57BL/6J(a/a) non-mutant lean mice.

**Conclusion:**

TZD administration may influence ovarian function via numerous diverse mechanisms that may or may not be directly related to insulin/IGF signaling.

## Background

Polycystic ovary syndrome (PCOS) is a reproductive disorder characterized by chronic anovulatory ovarian follicles and hyperandrogenism, often associated with obesity. It is the major cause of anovulatory infertility, affecting approximately 5–10% of women of reproductive age. PCOS is one of several health disorders associated with Metabolic Syndrome (Syndrome X), or subclinical insulin resistance. A decrease in insulin sensitivity leads to a compensatory increase in insulin secretion to maintain normal glucose tolerance. Hyperinsulinemia is thought to be a major contributing factor to the hyperandrogenism and ovulatory dysfunction in PCOS [1,2].

It has become common practice to treat women with PCOS with insulin-sensitizing drugs. The two classes of insulin-sensitizing agents currently used most often in clinical practice are biguanides (e.g. metformin) and thiazolidinediones (TZD), which include rosiglitazone and pioglitazone. These classes of drugs act via different mechanisms which are not thoroughly understood. In general, metformin reduces hepatic glucose synthesis leading to a direct reduction in insulin secretion [3]. TZD are ligands for the peroxisome proliferator-activated receptor γ (PPARγ), which enhances transcription of a variety of genes that collectively promote glucose disposal.

Metformin has been much more widely used and studied in women with PCOS than have TZD. Women treated with metformin have significantly improved spontaneous ovulation and ovulatory response to gonadotropins, diminished androgen levels, lowered blood pressure, and enhanced fertility [4-7]. Limited studies with TZD have shown similar results [8,9].

Although overall improvement in symptoms appears to be similar with metformin or TZD, these drugs clearly act via distinct mechanisms. For example, TZD (troglitazone), but not metformin, reduced androgen levels by directly suppressing 3β-hydroxysteroid dehydrogenase and cytochrome P450 17α-hydroxylase, two key enzymes in the androgenic pathway [10]. Moreover, insulin and insulin-like growth factor cellular signaling were differentially altered in ovarian cells from women with PCOS, but after TZD treatment cell signaling was similar to cells from non-PCOS women [11]. In that study, insulin receptor substrate (IRS)-1 and -2 expression by ovarian granulosa cells from women with PCOS was altered relative to cells from women without PCOS; IRS-1 expression was higher and IRS-2 expression was lower in PCOS cells. However, after troglitazone treatment *in vitro*, IRS-1 expression was reduced and IRS-2 expression was enhanced bringing the levels of expression similar to those found in non-PCOS cells [11].

Mice heterozygous for the Lethal Yellow (LY) mutation at the agouti locus exhibit distinct characteristics including yellow coat color, adult onset obesity, and accelerated reproductive senescence [12]. Moreover, as these mice age, they progressively develop insulin resistance and hyperinsulinemia [13], hyperleptinemia [14], and central leptin resistance [15]. Diminishing ovarian function in aging LY mice is directly related to increasing obesity and the progression of altered metabolic regulation [16]. LY mice provide a useful animal model for investigating the effects of insulin-sensitizing drugs on ovarian gene expression in a hyperinsulinemic/insulin resistant state. The current study tested the hypothesis that chronic treatment of aging LY mice with the insulin-sensitizing drug, pioglitazone, alters the ovarian expression of genes that may be involved in insulin signaling or other aspects of ovarian function.

## Methods

### Animals

The study was approved by the Augustana College Animal Care and Use Committee. Black (BL; C57BL/6J a/a) and Lethal Yellow (LY; C57BL/6 A^y^/a) mice from the Augustana College Biology Department breeding colony were used for the study. Founder mice were originally obtained from Jackson Laboratory (Bar Harbor, ME, USA). Mice were fed maintenance diet (Harlan Teklad, Madison, WI, USA) and fresh water *ad libitum*, and housed in groups of four mice per cage on a 14:10 light/dark cycle with lights on at 0600 as described previously [16]. Beginning at 120-days of age, female LY mice received daily oral doses of either 0.01 mg pioglitazone (n = 4 mice) or an equal volume of vehicle (DMSO; n = 4 mice) for 60 consecutive days. This dosage of pioglitazone was chosen to approximate the dosage used clinically in humans after adjustment for body mass, i.e. 30 mg daily for a 50–70 kg adult.

At the end of the treatment regimen, the now 180-day old treated LY mice and untreated 180-day old C57BL/6J non-mutant control mice (n = 2) were weighed and then euthanized by cervical dislocation after overnight fasting. For all mice, blood was collected by heart puncture, glucose concentrations were measured immediately on whole blood, and serum was processed and stored at -20C for later analyses. Both ovaries were removed and carefully trimmed of adhering fat and connective tissue. One ovary from each animal was quickly placed in RNALater (Ambion, Austin, TX) for subsequent RNA extraction. The contralateral ovary was placed in cell lysis buffer (Sigma), homogenized, centrifuged, and protein extracts were stored at -80C for later Western blot analysis.

### Hormone and glucose assays

Glucose was measured in fresh whole blood using a Freestyle (TheraSense, Alameda, CA) glucometer, linear range = 20–500 mg/dL. Leptin concentrations were measured in serum in a single run using a homologous Mouse Leptin RIA kit (Linco Research, St. Louis, MO, USA) as described previously [16]. The interassay CV was 3.3%. Insulin was measured using the Mercodia Ultrasensitive Mouse Insulin ELISA (ALPCO Diagnostics, Windham, NH). The sample volume was 5 μl. Intraassay CV was 6.2% and interassay CV was 5.1%.

### RNA extraction

RNA was extracted as described [17]. Each ovary was homogenized in 1 ml TRI reagent (Molecular Research Center, Cincinnati, OH). Sodium acetate and bromochloropropane were mixed with the homogenate, the sample was incubated on ice for 15 min, and then centrifuged to separate the phases. The aqueous phase containing RNA was removed and purified on an Rneasy column (Qiagen, Valencia, CA). The sample was treated with an on-column RNase-free DNase to remove any potentially contaminating genomic DNA. Total RNA was eluted from the column. The RNA concentration and purity were calculated using the RNA 6000 Nano LabChip in an Agilent Bioanalyzer. The RNA was stored at -70°C prior to processing for DNA microarray analysis.

### Microarrays

CodeLink Whole Mouse Genome Bioarrays (GE/Amersham, Piscataway, NJ, now Applied Micrarrays, Tempe, AZ) were used for the analysis of differential gene expression in this study. These microarrays contain 33,000 single-stranded 30-mer oligonucleotide probes for mouse genes and transcribed sequences. Biotinylated complementary RNA (cRNA) probes were synthesized from the extracted RNA samples per company directions as previously described [17] using CodeLink Expression Assay Reagent Kit (GE-Amersham Biosciences). Individual samples were run on separate microarrays (n = 4/LY treatment group; n = 2 for BL controls); no samples were pooled. The biotinylated cRNA was fragmented and hybridized with the DNA microarray slides for 18 hours at 37°C. The hybridized slides were washed and incubated with streptavidin-Alexa Fluor 647 (Molecular Probes) to label the cRNA, and washed again. An Axon GenePix Scanner was used to scan the microarrays. GenePix Pro software (MDS, Inc., Toronto, ON) was used to acquire and align the microarray image. CodeLink software (Applied Microarrays, Tempe, AZ) applied the background correction. GeneSpring 7.0 software (Agilent, Santa Clara, CA) was used to normalize the expression of each gene to the median gene expression and to normalize each slide to the the 50^th ^percentile of gene expression. Statistical analysis of the data was performed using GeneSpring 7.0 (Agilent). The p value was set at 0.05 for the t test. Multiple testing correction used the Benjamini and Hochberg False Discovery Rate. Approximately 5% of the genes would be expected to pass this restriction by chance with this test.

### Real time RT-PCR

Real time RT-PCR was used to confirm differential gene expression of resistin-like molecule alpha (RELMα). Attempts to confirm three additional genes, leptin, ARP6, and angiopoietin-like 4, were inconclusive and could not be repeated due to depletion of the RNA samples. Pre-designed primers and fluorescent (FAM) labeled minor groove binding probe were obtained from Applied Biosystems (Foster City, CA). Real time RT-PCR was carried out with TaqMan Gold RT-PCR reagents (Applied Biosystems) as described [17]. Changes in expression of RELMα were calculated relative to an endogenous control (GAPDH). An RNA concentration-response validation curve was carried out to determine the concentration of RNA to add to the RT-PCR reaction. All samples were run in duplicate, n = 3. The qBase software [18] was used to analyze the data from the real time RT-PCR reaction. The qBase program uses a delta Ct (threshold cycle) relative quantitation model with PCR efficiency correction and multiple reference gene normalization. The real time RT-PCR data were analyzed by t-test with GraphPad Prism software (GraphPad Software, San Diego, CA).

### Immunocytochemistry

Paraffin sections from 180-day old untreated LY mice that were available from previous studies were used to confirm the presence of RELMα in mouse ovary since all ovaries from the present study were used for other purposes. VectaStain Rabbit IgG Elite ABC kit (Vector Laboratories, Inc., Burlingame, CA) was used for immunocytochemistry. Sections were de-paraffinized, re-hydrated, and blocked with 3% hydrogen peroxide in methanol for 15 min. Antigen retrieval pre-treatment was performed by sequential microwaving in citrate buffer. After washing, sections were blocked with blocking serum and rinsed. Sections were incubated with primary antibody (anti-RELMα; Upstate/Chemicon, Temecula, CA) for 1 h at RT, washed thrice, and incubated with biotinylated second antibody for 30 min. at RT. Slides were washed and incubated with VectaStain ABC reagent for 30 min. at RT. DAB substrate was used for colorimetric antigen detection, and sections were counterstained with hematoxylin. Each slide contained three serial tissue sections, one of which was always incubated without primary antibody to serve as a control. No staining was observed in any control sections.

### Western blotting

Samples and pre-stained standards (Bio-Rad MagicMark XP, Invitrogen) were loaded into precast Tris-HCl mini-gel (Bio-Rad). Gels were run using constant current of 15 mA for the stacking gel and 23 mA for the running gel. Proteins were transferred with constant voltage to 7 × 8.5 cm Biotrace PVDF Transfer Membranes (VWR International, Inc) for 1 hour at 120 v. Following transfer, the membranes were blocked for 1 hour at room temperature with 5% ECL blocking agent (GE Healthcare) in TBS-Tween. The membrane was then incubated overnight at 4C in primary antibody (same as used for immunocytochemistry) diluted in 1% ECL blocking/TBS-tween. Following three times five minute washes with TBS-tween, the membrane was incubated for 1 hour at room temperature with horse radish peroxidase conjugated secondary antibody (R&D Systems, Inc). The membrane was washed three times five minute with TBS-tween before development with Lumi-Light PLUS Western Blotting Substrate (Roche Diagnostic Corp). Bands were visualized by enhanced chemiluminescence. Relative band intensities were compared by determining the ratio of the area densities of RELMα to actin bands for each lane using Image-Pro Plus^® ^software (MediaCybernetics). Ratio means were compared between treatment groups by t-test.

## Results

Oral pioglitazone treatment for 60 days in LY mice did not significantly alter body weight, or fasting serum glucose, insulin, or leptin levels (Table 1), although fasting insulin tended (p = 0.09) to be slightly lower in pioglitazone-treated mice. Body weight, insulin, and leptin levels were all higher in LY mice than in age-matched, untreated BL mice.

**Table 1:** Body weight (BW) and fasting serum concentrations of leptin, insulin, and glucose in 180-day old vehicle- and pioglitazone-treated LY mice and untreated BL control mice.

	Vehicle	Pioglitazone	Untreated C57BL/6J
BW (g)	34.8 ± 5.3 ^a^	34.8 ± 4.4 ^a^	24.1 ± 0.4 ^b^
Leptin (ng/mL)	41.0 ± 11.4 ^a^	43.5 ± 12.4 ^a^	6.7 ± 2.4 ^b^
Insulin (ng/mL)	2.09 ± 0.19 ^a^	1.65 ± 0.35 ^a^	0.85 ± 0.05 ^b^
Glucose (mg/dL)	138 ± 10 ^a^	129 ± 3 ^a^	122 ± 3 ^a^

Means ± SD, n = 4 per group. Letter superscripts denote significant differences among groups (ANOVA, Duncans test).

Of 3.3 × 10^4 ^genes on the array, approximately 1500 showed a significant (p < 0.05) difference in expression. Unidentified genes and expressed sequence tags (EST) were removed from further analysis. In addition, those genes whose expression was less than 0.2 relative intensity units (the limit of sensitivity) in both control and treatment groups were excluded. After these exclusions, 27 genes representing a diversity of function exhibited a 2-fold or greater significant (p < 0.05) difference in expression level between groups (Table 2). Genes with reduced expression in pioglitazone-treated mice included leptin, angiopoietin, angiopoietin-like 4, Foxa3, and tenascin. Conversely, RELMα, prostaglandin (PG) D receptor, and PGE1 receptor all exhibited elevated expression in pioglitazone-treated LY mice. The data set for these DNA microarrays has been deposited at the National Center for Biotechnology Information Gene Expression Omnibus [19] as recommended by Minimum Information About a Microarray Experment (MIAME) standards [20] and can be accessed through accession number GSE8806.

**Table 2:** Genes with two-fold or greater (p < 0.05) differential expression after pioglitazone treatment.

**Accession #**	Δ^1 ^(pioglitazone)	Δ^2 ^(C57BL/6J)	**Gene name**
NM_008493.3	0.28	0.17	leptin (Lep)
NM_009640.2	0.35	0.65	angiopoietin (Agpt)
NM_177839.2	0.44	0.61	tenascin N (Tnn)
CF906297.1	0.46	0.37	formyltetrahydrofolate synthetase domain 1
AK083494.1	0.46	0.72	ubiquitin specific protease 29
NM_008260.1	0.47	0.54	forkhead box A3 (Foxa3)
CK329907.1	0.49	0.45	aconitase 2, mitochondrial
AK005115.1	0.50	0.85	zinc-finger RNA-binding domain containing 1
NM_025914.1	0.50	0.65	actin-related protein 6 homolog, ARP6
NM_020581.1	0.50	0.88	angiopoietin-like 4 (Angptl4)
NM_008962.2	2.00	2.54	prostaglandin D receptor (Ptgdr)
NM_178779.2	2.00	2.40	ring finger protein 152
BC025027.1	2.00	1.59	similar to phospholipase C, epsilon
NM_021462.2	2.02	1.92	MAP kinase-interacting ser/thr kinase 2
NM_134249.3	2.04	2.05	T-cell immunoglobulin/mucin domain 2 (Timd2)
NM_011904.1	2.05	1.84	tolloid-like 2 (Tll2)
AK053616.1	2.07	1.77	prostaglandin E receptor 1
NM_009188.1	2.08	2.52	transcriptional regulator, SIN3B (Sin3b)
BY482739.1	2.08	1.54	membrane associated guanylate kinase interacting-1
AI604236.1	2.32	1.50	E74-like factor 1
NM_021447.1	2.50	2.05	ring finger protein 30 (Rnf30)
**NM_020509.2**	**2.62**	**1.04**	**resistin-like alpha (RELMα)**
NM_008830.1	2.82	2.18	ATP-binding cassette, subfamily B, member 4
NM_148937.1	3.33	1.81	phospholipase Cδ 4
AF223416.1	3.55	1.85	cardiac triadin isoform 2 mRNA
NM_198864.2	3.58	1.91	SLIT and NTRK-like family, member 3 (Slitrk3)
BB393194.2	5.22	0.84	dipeptidylpeptidase 10

Vehicle-treated LY mice (controls) were assigned a level of expression of 1.0 for each gene Relative expression of pioglitazone-treated LY mice (Δ^1^) and untreated BL non-mutant mice (Δ^2^) compared with control mice is expressed as the fold-difference of the mean level of expression of four (vehicle- and pioglitazone-treated) or two (untreated C57BL/6J) animals per group.

Relative expression of these 27 genes in untreated BL mice (n = 2) was included for comparison (Table 2). For most genes, pioglitazone tended to alter expression towards levels seen in the BL control mice. Exceptions were RELMα and dipeptidypeptidase 10, whose expression was similar in vehicle-treated LY mice and untreated BL mice, but greatly elevated in pioglitazone-treated LY mice.

**Figure 1:**
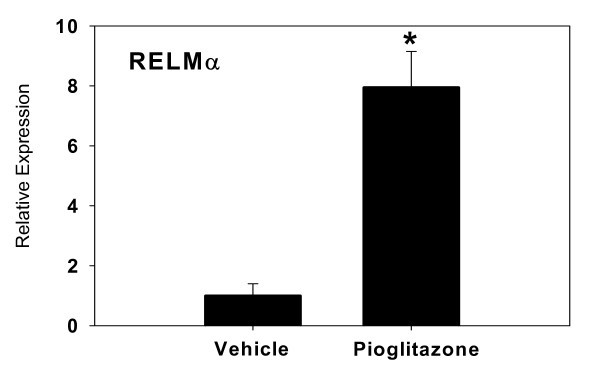
**Relative increase in expression of RELMα by Real Time RT-PCR.** Bars represent mean ± SD, in relative gene expression calculated relative to endogenous GAPDH expression. An RNA concentration-response validation curve was carried out to determine the concentration of RNA to add to the RT-PCR reaction. All samples were run in duplicate, n = 3 animals per group.

**Figure 2:**
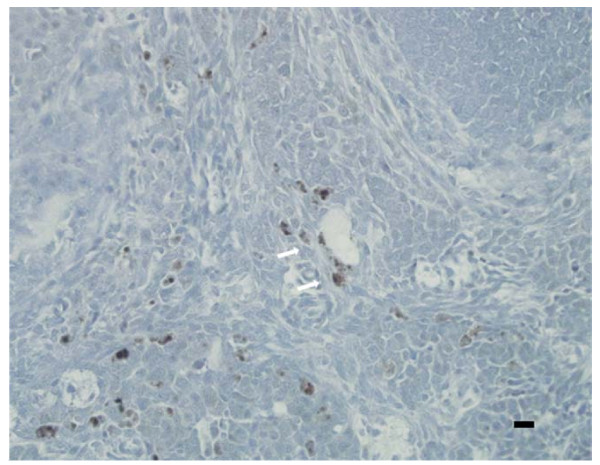
**Immunolocalization of RELMα in a untreated 180-day old LY mouse ovary.** VectaStain Rabbit IgG Elite ABC kit (Vector Laboratories, Inc., Burlingame, CA) was used on paraffin sections. Sections were incubated with primary antibody (anti-RELMα; Upstate/Chemicon, Temecula, CA) for 1 h at RT, washed thrice, and incubated with biotinylated second antibody for 30 min. at RT. DAB substrate (brown precipitate, arrows) was used for antigen detection, and sections were counterstained with hematoxylin. Scale bar = 25 μm (lower right).

**Figure 3:**
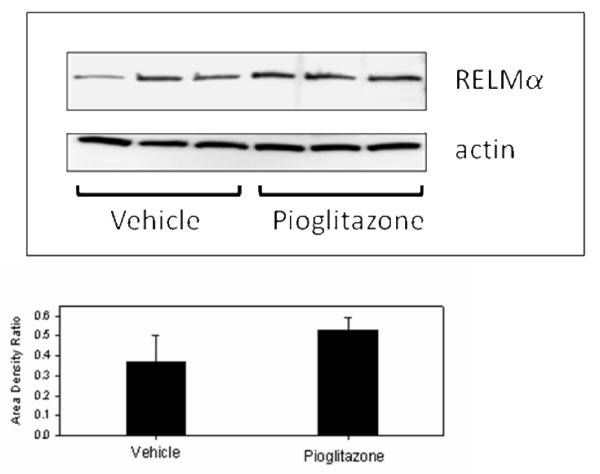
**Western blot of RELMα immunoactivity in vehicle- and pioglitazone-treated LY mice.** Beta-actin immunoactivity was used as a loading control. Bar graph depicts the mean (+ SD) ratio of intensity of RELMα-to-actin bands. There was no significant difference in band intensity between groups.

Expression of RELMα in the ovary has not been previously reported. Therefore microarray results were verified by Real Time RT-PCR. The difference in expression of RELMα by RT-PCR was nearly eight-fold (Figure 1), confirming a significant increase in mRNA levels in pioglitazone-treated mice. Since gonadal fat is a known source of RELMα [21], we sought to confirm that the RELMα expression was within the ovary proper. RELMα protein was detected in mouse ovarian sections from untreated age-matched LY mice by immunocytochemistry, confirming that RELMα was expressed within the ovary rather than in adhering fat tissue. RELMα primarily localized in perivascular cells, which on the basis of morphology and distribution were likely to be macrophages, although this was not confirmed (Figure 2). Western blot analysis of protein extracts of vehicle- and pioglitazone-treated LY mice did not reveal a significant difference in protein expression (Figure 3).

Because of limited quantities of RNA extracts available after microarray analyses, no other genes could be verified by RT-PCR. Attempts to confirm three additional genes yielded inconclusive results, which could not be repeated due to depletion of the samples.

## Discussion

We have previously shown that progressive obesity in aging LY mice is associated with a concomitant diminution in ovarian function, which is paralleled by acquired insulin and leptin resistance [16]. To our knowledge, this is the first report demonstrating that prolonged in vivo exposure to TZD alters intraovarian gene expression in an aging obese mouse model. The altered expression of numerous genes involved in diverse cellular functions such as cell signaling, cell proliferation and survival, cell adhesion, and differentiation suggest that the positive effects of TZD on ovarian function observed in PCOS patients may be multifactorial.

TZD are primarily characterized as ligands for the PPARγ. However, pioglitazone may also activate PPARα, which may explain its broader spectrum of clinical action relative to other TZD [22]. Ligand binding of either of the two PPARγ isoforms results in heterodimerization with a retinoid X receptor (RXR), followed by the PPARγ/RXR complex binding to and activating PPAR response elements on target genes [23]. In general, PPARγ activation in adipose cells has anti-angiogenic actions and suppresses the synthesis and secretion of proinflammatory and proinsulin resistant adipokines [24].

PPAR are widely expressed in vascular and inflammatory cells [25]. In the ovary, PPARγ are localized in granulosa cells of maturing follicles [26,27] and in thecal and stromal macrophages [28]. In contrast, PPARα are expressed throughout thecal and stromal regions of rodent ovaries [26,27] consistent with vascular and peri-vascular expression.

TZD actions in the ovary have not been widely investigated. Troglitazone suppressed LH and insulin-stimulated androgen production by isolated porcine thecal cells [29]. Moreover, in human follicular fragments, TZD (pioglitazone and rosiglitazone) modestly inhibited testosterone and estradiol production, but enhanced progesterone and IGFBP-1 production [30]. These results in isolated ovarian cells imply a direct action of TZD on steroidogenesis, although the mechanisms remain unclear. In addition, troglitazone suppressed inducible nitric oxide synthase (NOS2) in mouse ovarian macrophages isolated from preovulatory ovaries [28]. Perivascular/anti-inflammatory actions of TZD in the ovary may also alter ovarian function.

Among genes responding to pioglitazone with altered expression were several that participate in vascular remodeling. An increase in angiogenesis and stromal blood flow are characteristic of PCOS [31,32]. Notably, leptin, angiopoietin, angiopoietin-like 4, and RELMα were among the genes whose expression was altered most by pioglitazone treatment. The three-fold reductions in leptin and angiopoietin mRNA expression in the current study would be consistent with reduced angiogenesis. In contrast, RELMα has generally been reported to have angiogenic actions [33], so the significant increase in its gene expression in the current study may appear inconsistent. Angiopoietin-like 4, has been reported to be both angiogenic [34] and anti-angiogenic [35].

A novel finding was the intraovarian expression of RELMα and its up-regulation by pioglitazone. Resistin and RELMα are often co-regulated [36]. Resistin has been shown to have significantly lower expression in mouse models of obesity, e.g. *ob/ob *and *db/db *mice, but is stimulated by PPARγ agonists [37]. Therefore the increased expression of RELMα by pioglitazone in the current study would be consistent, although resistin expression was not significantly altered by pioglitazone in this study. RELMα, also known as hypoxia-induced mitogenic factor (HIMF) or FIZZ1, has not previously been shown to be significantly expressed in the ovary, although it is expressed in high levels in gonadal fat of lactating mice [21]. RELMα is reported to be primarily associated with adipose tissue, specifically localized in the vascular fraction [36]. The present study demonstrates RELMα protein expression in perivascular cells within the ovary, which are consistent in appearance and distribution with macrophages, known targets of TZD. However, RELMα protein levels on Western blot were not siginificantly greater in pioglitazone-treated ovaries. Considering the proximity of the immunoactive cells to blood vessels, it is possible that if RELMα is secreted by these cells it could be primarily carried off in the circulation and would not show accumulation in the tissue.

## Conclusion

Prolonged oral administration of pioglitazone to aging obese LY mice altered the ovarian expression of a several genes. The results suggest that PPARγ may mediate diverse actions in the ovary, and that the positive effects of TZD observed in women with PCOS might involve mechanisms not directly related to insulin sensitivity. Further research is needed to investigate the wide range of potential regulatory actions of PPARγ agonists in normal ovarian physiology and PCOS.

## Authors' contributions

JB and MD conceived and designed the study. MW carried out the treatments and tissue collection, and prepared preliminary data summaries. KE performed RNA extractions and microarray analyses, and performed statistical analyses on microarray data. JB and MD performed final data analysis, and JB drafted the manuscript.
